# Muscle Strength and Phase Angle Are Potential Markers for the Efficacy of Multidisciplinary Weight-Loss Program in Patients with Sarcopenic Obesity

**DOI:** 10.3390/jcm13175237

**Published:** 2024-09-04

**Authors:** Amelia Brunani, Ettore Brenna, Antonella Zambon, Davide Soranna, Lorenzo Maria Donini, Luca Busetto, Simona Bertoli, Paolo Capodaglio, Raffaella Cancello

**Affiliations:** 1 Laboratory of Biomechanics, Rehabilitation and Ergonomics, IRCCS, Istituto Auxologico Italiano, 28824 Piancavallo Verbania, Italy; p.capodaglio@auxologico.it; 2 Biostatistic Unit, IRCCS, Istituto Auxologico Italiano, 20149 Milan, Italy; e.brenna@auxologico.it (E.B.); antonella.zambon@unimib.it (A.Z.); d.soranna@auxologico.it (D.S.); 3 Department of Statistics and Quantitative Methods, University of Milan-Bicocca, 20126 Milan, Italy; 4 Department of Experimental Medicine, Sapienza University, 00161 Rome, Italy; lorenzomaria.donini@uniroma.it; 5 Department of Medicine, University of Padova, 35122 Padua, Italy; luca.busetto@unipd.it; 6 Obesity Unit, Laboratory of Nutrition and Obesity Research, Department of Endocrine and Metabolic Diseases, IRCCS, Istituto Auxologico Italiano, 20149 Milan, Italy; s.bertoli@auxologico.it (S.B.); r.cancello@auxologico.it (R.C.); 7 Department of Surgical Sciences, Physical Medicine and Rehabilitation, University of Torino, 10126 Torino, Italy

**Keywords:** phase angle, skeletal muscle mass, muscle strength, sarcopenic obesity, weight loss, obesity

## Abstract

**Background/Objectives**: Traditional weight-loss methods often result in the loss of both fat and muscle mass. For individuals with sarcopenic obesity (SO), additional muscle loss can exacerbate sarcopenia, leading to further declines in muscle strength and function, ultimately worsening quality of life. To mitigate this risk, weight-loss strategies should emphasize the preservation and building of muscle mass through adequate protein intake and tailored resistance training. This study aimed to evaluate changes in SO status following a 4-week multidisciplinary weight-loss intervention program in hospitalized patients with obesity. **Methods**: This study included adult patients with obesity (BMI > 30 kg/m^2^, aged 18–90 years). The SO diagnosis was performed using the handgrip strength (HGS) test and skeletal muscle mass (SMM) by bioelectrical impedance analysis (BIA) according to ESPEN/EASO-2022 guidelines. **Results**: A total of 2004 patients were enrolled, 64.8% female, with a mean age of 56 (±14) years and a BMI of 40.7 (±6.48) kg/m^2^. SO was present in 9.38% (188 patients) at baseline. At discharge, 80 patients (42.55%) were no longer classified as sarcopenic and showed significant improvements in HGS. The likelihood of resolving SO was not modified in patients with only phase angle (PhA) improvement (*p*-value = 0.141). Patients with HGS increment had a 65% probability to be No-SO at discharge and this probability, with the concomitant PhA increment, rose to 93% (*p*-value < 0.0001), indicating that functional changes and good nutrition status are crucial in improvement of SO. Muscle mass (MM) and SMMI remained unchanged in the studied cohort. **Conclusions**: Improvements in HGS and the PhA are potential markers for the efficacy of weight-loss programs tailored to patients with SO. These findings suggest that specific interventions focusing on these markers could be beneficial in managing SO patients.

## 1. Introduction

In patients with obesity, weight loss produces different metabolic benefits such as a reduction in glycemic and lipid profile, lower blood pressure with a reduction in the worsening of clinical complications, and a reduction in cardiovascular risks. These effects are mainly due to a reduction in fat mass (FM), but are frequently associated with a certain degree of fat-free mass (FFM) loss, which includes skeletal muscle mass (MM), and typically represents 20–40% of the body weight loss [1]. Furthermore, muscle mass (MM) is an independent marker of metabolic health, and any loss of MM, even if unintentional, could impair quality of life and lead to a decline in physical function [1]. Thus, it is crucial to assess body composition in patients with obesity undergoing weight loss. Bioelectrical impedance analysis (BIA) is actually a widely used technique in body composition analysis, particularly for assessing the hydration, FFM, and MM of the body [2]. The base for the body composition analysis by BIA is the phase angle (PhA) derived from the relationship between the resistance (Rz) and reactance (Xc) of the body [3]. The PhA provides insights into the body’s cellular health and integrity and could be considered as a potential indicator of various health conditions when used in clinical settings for prognostic outcomes [4,5].

Due to its potential clinical relevance, the PhA is now considered an underestimated parameter useful in clinical practice. In individuals living with obesity, the PhA is influenced by various factors, including sex, age, body composition [such as fat mass (FM) and fat-free mass (FFM)], hydration status, and nutrition state. In fact, the PhA is a well-known biomarker in assessing metabolic health, providing insights into cellular integrity, nutritional status, and overall body composition. In metabolic health, a higher PhA is often correlated with a better nutritional status, reflecting adequate protein stores, muscle mass, and overall body composition. Lower PhA values have been associated with conditions such as metabolic syndrome, type 2 diabetes, and obesity, indicating a higher risk of complications or poorer outcomes, also making it valuable in monitoring disease progression. Actually, the PhA is increasingly used as a prognostic tool in clinical settings. It can help predict outcomes in patients with chronic illnesses, including cardiovascular diseases, cancer, and liver disease. Changes in the phase angle over time can reflect the effectiveness of interventions aimed at improving metabolic health, such as diet, exercise, or medical treatment. An increasing phase angle suggests improvement in cellular health and global metabolic function [3,4,5].

In addition, the PhA positively correlates with various markers of muscle health and overall well-being, such as muscle area, muscle circumference, muscle echo intensity, serum protein levels, quality of life, and physical performance strength [6,7]. Interestingly, in the context of SO, a condition of the simultaneous presence of reduced muscle mass and increased adiposity, the PhA has been studied as an indicator of MM quality and cellular health [8]. A lower PhA in patients with SO is associated with negative health outcomes, reflecting a decreased muscle function and integrity [9,10]. Individuals with SO and a lower PhA have been found to be at increased risk of mortality, show poorer physical performance, and higher incidence of chronic diseases, such as cardiovascular disease, diabetes, and metabolic syndrome [11,12]. The PhA, when combined with other measures such as inflammation markers, can provide a more comprehensive assessment of the health status of individuals with SO [13].

Weight-loss interventions that prioritize the preservation of MM, such as a combination of resistance training and adequate protein intake, are more likely to result in favorable changes in body composition in older adults with obesity [6,7]. Recent studies have indicated that during weight loss induced by bariatric surgery, factors affecting cellular function, such as oxidative damage, and variables related to physical exercise, such as muscle strength and aerobic fitness, may also impact PhA values [8,9,10,11]. Koehler et al. [10] observed a moderate correlation between the PhA and serum transthyretin (TTR) concentrations during rapid weight loss post-RYGB surgery in women. These findings collectively suggest that reduced PhA values in patients undergoing RYGB and sleeve gastrectomy (SG) may indicate a concurrent decline in visceral protein status, lean body mass, and potentially protein nutritional status [10,12]. Considering that the PhA represents a functional parameter of MM related to the disability index, the potential change in the PhA could be used as a marker of changes in functional aspects, especially in patients with SO [13]. At present, few data are available on the PhA variations during weight loss induced by hypocaloric diet and physical activity (multidisciplinary rehabilitation program) in a selected population of patients with SO. We decided to conduct a study to determine whether weight loss, during a 4-week multidisciplinary weight-loss program, worsened or improved sarcopenia in patients with sarcopenic obesity (SO). Additionally, we aimed to assess a predictive model to estimate the probability of improving SO at hospital discharge.

## 2. Materials and Methods

### 2.1. Patients

**Figure 1:**
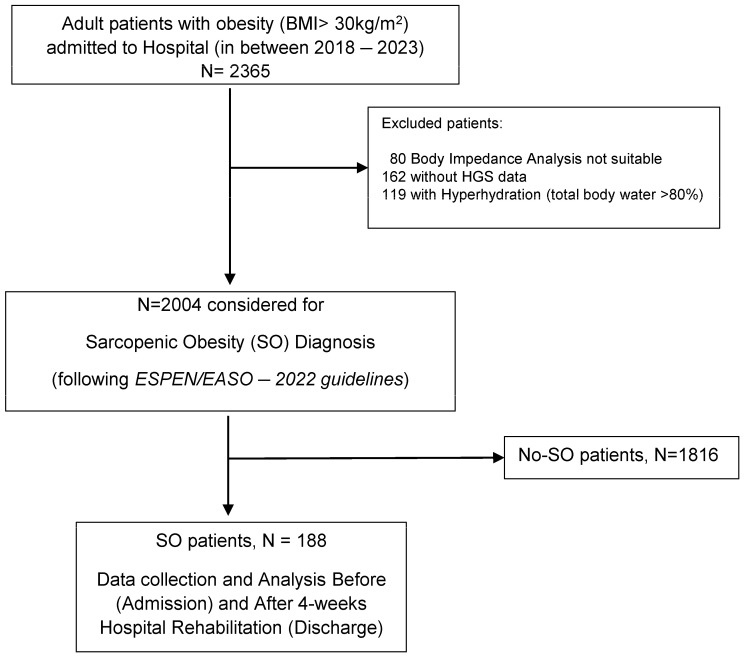
Flow chart of patient enrollment, exclusion, and analysis.

We conducted the study at the Istituto Auxologico Italiano, IRCCS (Scientific Institute for Research, Hospitalization, and Healthcare), Piancavallo (Verbania, Italy), including consecutively admitted in-patients from April 2018 to December 2021. These patients were admitted for diagnostic evaluation and a 4-week multidisciplinary rehabilitation program aimed at addressing obesity through metabolic, nutritional, and psychological interventions. We included patients of both sexes, aged 18 to 90 years, with a BMI ≥ 30 kg/m^2^, and suitable for body composition assessment. We excluded patients that were not eligible for body composition analysis (such as patients with pacemaker implants and/or leg/arm amputation) and/or not able to perform the handgrip test. The analysis was focused on patients who completed the program and underwent a repeated evaluation within the three days leading up to the hospital discharge. The summary of the patient cohort is reported in Figure 1. This study was approved by the institutional ethics committee of Istituto Auxologico Italiano (details available at https://www.auxologico.it/ricerca-formazione/comitato-etico, accessed on 3 September 2024) with the approval ID number 2021_05_18_07. All procedures were conducted as part of routine hospital care, and each patient provided written informed consent for the use of their data for research purposes upon admission. The research adhered to the principles outlined in the 1964 Helsinki Declaration, its subsequent amendments, and the ethical guidelines set by the institutional and national research.

### 2.2. Anthropometric Parameters

Body weight (kg) and body height (meters) were measured with precision to the nearest 0.1 kg and 0.5 cm, respectively. A mechanical column scale (Scale-Tronix, Wheaton, IL, USA) and a stadiometer (Scale-Tronix, Wheaton, IL, USA) were used for these measurements. Body mass index (BMI) was then calculated by dividing the body weight by the square of the height (kg/m^2^). The waist circumference (cm) was measured with non-elastic tape at the level of the umbilicus.

### 2.3. Body Composition

Body composition analysis was performed by a single-frequency bioimpedance analyzer (BIA 101, Akern^®^, Pisa, Italy) as previously described [2]. Prior to the measurement, each subject removed their clothing and any metal jewelry and rested in a supine position for five minutes to allow for fluid equilibration in the body. Before each testing session, the impedance analyzer was calibrated using a reference circuit with a known impedance value (Rz = 380 Ω and Xc = 47 Ω) with a maximum allowable error of 1%. The mean coefficient of variation for within-day measurements in steady-state conditions was 1%, and for intra-individual measurements, it was 3%. The inter-operator variability had a mean coefficient of variation of 2%. These values indicate the level of measurement precision and reliability for the bioimpedance analyzer used in the study. Individuals with hydration levels [calculated as the ratio between total body water (L) and fat-free mass (kg)] were excluded when exceeding 80% to avoid overestimation of FFM.

### 2.4. Skeletal Muscle Mass Index

We applied the following formula of Janssen et al. [14] to calculate skeletal muscle mass. The skeletal muscle mass index (SMMI) was the percentage of the ratio between the SMM and the total body weight in kg [(SMM/W) × 100].

### 2.5. Muscle Strength

Muscle strength was measured with the handgrip strength test (HGS), using a dynamometer (JAMAR^®^ (Lafayette Instrument Company, Lafayette, IN, USA) isometric dynamometer on both arms, dominant or not). Three repeated measurements were taken for both left and right hand and the mean values were calculated between the measures, as reported in [15].

### 2.6. Psychological General Well-Being Index (PGWBI) Questionnaire

The Italian version of the PGWBI questionnaire validated by Grossi et al. [16] was used as previously described [17]. The PGWBI scoring for quality of life (QoL) evaluation was considered as follows: “good” with a score >70; “normal” with a score between 60–70; and “poor” with a score < 60. Questionnaires not fully completed (with missing data in subscales sections and before/after the time of the study) were excluded from the analysis.

### 2.7. Criteria for Definition of SO

The ESPEN/EASO-SO consensus criteria [18] were applied, respecting in sequence, the following two steps of the diagnostic criteria: (1) muscle strength deficit assessed by HGS (<27 kg in men and <16 kg in women) and (2) SMMI deficit (<37% in men and <27.6% in women).

### 2.8. Multidisciplinary Weight-Loss Program

The intensive rehabilitation program lasted for a duration of 4 weeks. The multidisciplinary rehabilitation program included individualized nutritional intervention, psychological support, and supervised physical activity throughout the hospital stay. None of the patients received pharmacological prescriptions with the intention of achieving drastic weight reduction during the program. All patients received a balanced, hypocaloric Mediterranean diet consisting of three meals a day with 18–20% protein, 27–30% fat (of which <8% saturated fat), 50–55% carbohydrates (<15% simple sugars), and 30 g of fibers from fresh vegetables. Adherence to the 4-week protocol treatment was monitored by the nutritional service’s trained personnel. Under the supervision of a physiotherapist, two 60 min physiotherapy sessions were performed daily, consisting of personalized progressive aerobic training (e.g., walking, recline cycling, and arm ergometer exercises), postural control exercises, and progressive strengthening exercises. These supervised activities were designed to improve joint mobility and enhance cardiorespiratory fitness. Psychological counseling was an integral part of the program and focused on educating patients about the management of their emotions, eating behavior, and establishing control over their food intake.

### 2.9. Statistical Analysis

Continuous variables with normal distribution are represented as mean and standard deviation and those with a skewed distribution are represented as median and interquartile range [IQR]. Categorical variables are presented as absolute frequency and proportion. For the comparison between SO and non-SO patients of the continuous variables, the *T*-test (or Wilcoxon test) was performed, while for categorical, the Chi-square test (or Fisher test). The outcome variable of interest was categorical (two levels) sarcopenia status at discharge (SO or No SO). To identify the determinants of No SO state at discharge, we applied a logistic regression model including as covariates two dichotomous variables related to increments in HGS and the PhA and their interaction. We also included the following covariates suggested by clinicians: age, gender, FFM (%), FM (%), number of comorbidities, and weight change (%). From the model, we estimated the marginal predicted probabilities of ‘No SO state’ at discharge, for different combinations of HGS and PhA improvement adjusting for confounders, using the marginal standardization. This method allows inference of the total population from which data are drawn. Briefly, marginal probabilities reflect a weighted average over the distribution of the confounders and are equivalent to estimates obtained by standardizing to the total population [19].

## 3. Results

A total of 2004 patients were considered for this study and participated in the 4-week hospital rehabilitation program (Figure 1). The cohort was composed of 62% female patients, with a mean age of 56 (±14) years. The mean BMI was 42.9 (±6.5) kg/m^2^. Class I and II of obesity (i.e., BMI between 30 and 39.9 kg /m^2^) was present in 43% of the cohort and class III (BMI ≥ 40 kg/m^2^) in 57%. The mean FM was 48.3% (±6.8%), FFM 51.7% (±6.8%), and mean MM 32.9 kg (±8.7 kg). All patients had a deficit in SMMI while the muscle strength deficit was present in 188 patients and 33% of the sample had at least one comorbidity (out of these, 40% had osteoarthritis).

### 3.1. SO Diagnosis and Characteristics at Admission

When applying ESPEN/EASO SO diagnostic criteria, the frequency of patients with SO was found to be 9.38% (*n* = 188) (Figure 1). There was a lower proportion of male patients among those with SO compared to without SO diagnosis at admission (31% vs. 38%, *p* = 0.0576). Additionally, the SO patients were significantly older (mean age 64 ± 12 years vs. 56 ± 14 years, *p* < 0.0001). We observed a high prevalence of patients aged > 65 years (56.9%), while there was a mean BMI of 40.7 (±5.8) kg/m^2^ and 49.5% for the cohort in class I and II of obesity, and 50.5% in class III. The mean FM was 48.2% (±6.5%), the mean FFM was 51.3% (±6.5%), the mean MM 28.8 (±7.1) kg, and the PhA 4.14° (±0.83°). The PGWBI score was “Poor” in 49.7%, “Normal” in 23.5%, and “Good” in 26.8%. Males were older, more frequently in class III obesity, and with a better body composition with respect to females.

The gender-specific clinical characteristic of SO patients at admission are reported in Table 1.

### 3.2. SO Patients at Discharge

At the end of the multidisciplinary program, 42.6% (*n* = 80) out of 188 SO patients at admission resulted in not being sarcopenic (No SO _discharge_), while for 108 patients, the SO condition was unchanged (SO _discharge_). The clinical characteristics of No SO _discharge_ and SO _discharge_ patients are reported in Table 2. The HGS and FM (kg) mean values were statistically higher in No SO _discharge_ (*p*-value < 0.0001 and *p*-value = 0.020, respectively). The PhA was not significantly different (*p*-value = 0.564).

Table 3 reports, for both groups (No SO _discharge_ and SO _discharge_)_,_ the mean change (calculated as value at discharge minus value at admission) in the considered parameters and the *p*-values related to their comparisons. The PhA was not significantly different in the two groups (*p*-value = 0.0937). The No SO _discharge_ patients showed a higher increment for HGS and PGWBI score with respect to the SO _discharge_ patients (*p*-value < 0.0001 and *p*-value = 0.0054, respectively).

**Figure 2:**
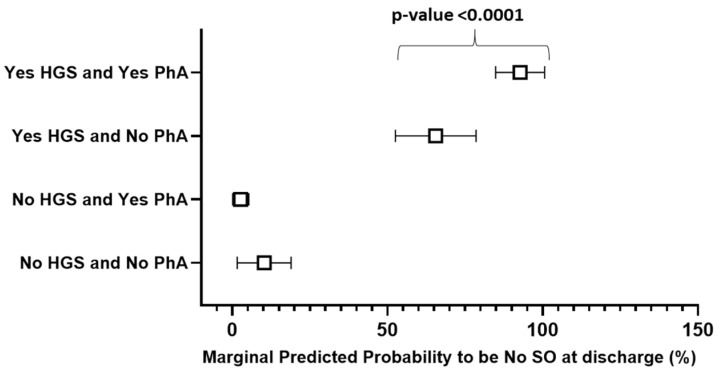
Population predicted probabilities of No SO state at discharge for different combinations of HGS and PhA changes (presence of change, Yes, absence of change, No).

Figure 2 shows the marginal predicted probability of the No SO state at discharge. Patients without an increment in both the PhA and HGS had a probability of 10%. When only the PhA increased, the probability did not change (*p*-value = 0.141). The HGS increment alone boosted the probability to 65% while in the presence of the PhA increment, the probability rose to 93% (*p*-value < 0.0001). When implementing the model by including the dichotomous variable SMMI delta changes instead of the variable HGS, we found that the interaction was not statistically significant (*p*-value = 0.2563).

## 4. Discussion

In this study, we observed that, during a multidisciplinary in-hospital weight-loss program, 42.8% of patients affected by SO improved their clinical condition. This result is mainly due to a recovery and a better HGS, which became more significant when associated with a positive increase in the PhA values. This is an important outcome in patients with SO and the monitoring of the changes in these two parameters can provide insights into the effectiveness of interventions aimed at improving body composition and overall health in this patient population.

The goal of rehabilitating individuals with obesity is not only to achieve weight loss, but also to promote functional muscle recovery and strengthening in those who have been weakened by obesity-related factors. It is known that weight-loss programs can unintentionally lead to a loss of skeletal muscle mass [1,20]. Therefore, it is essential to monitor the onset and progression of SO during weight-loss programs, as we have previously recommended in the serial monitoring of the PhA to preserve muscle or body cell mass (BCM) and restore normal fluid distribution [3]. In SO patients, after the rehabilitation program, we observed an improvement that resulted in 42.6% of the patients recovering from the SO condition. The primary factor responsible for this recovery was the improvement in the HGS, while the PhA did not change significantly after one month of rehabilitation. Interestingly, the SO group at discharge showed unchanged PhA values, whereas the No SO group at discharge exhibited a positive trend. This effect is confirmed by the logistic regression analysis where an increase in the PhA values, associated with an increase in HGs, increased the probability of the No SO state at discharge.

The significance of these observations needs to be investigated, in order to be explained. We can speculate that the efficacy of the rehabilitation program, even in a short time, resides in improving the quality of muscle mass rather (HGS) than its amount, leading first to a functional recovery. In fact, we know that in patients with obesity, the risk of increasing the loss of SMM during a weight management program is present with the probability of worsening SO. A ‘therapeutic’ weight loss [1], obtained with a hypocaloric diet + physical activity, might be considered as the combination of FM loss with a very slow rate of SMM loss, or unchanged SMM amount. On the other side, it was previously demonstrated that adding physical activity preserves or slightly improves muscle quality during weight loss, as measured by muscle attenuation and the intermuscular fat percentage, a measure of intramyocellular lipid content [6]. Since the first improvement was observed in HGS, it is tempting to speculate that a short-term weight loss with a balanced Mediterranean diet (with a mean of 18% of protein intake) could reduce the intra-muscular adipocytes (IMATs), improve the protein breakdown, and consequently the muscular function even in the absence of an SMM increase. These aspects deserve future investigations. HGS is a simple, non-invasive, and reliable measure that can provide valuable information about a patient’s muscle health and its use, alongside other assessments to monitor the effectiveness of interventions and to guide treatment decisions, should be encouraged in clinical settings.

Previous studies showed that a PhA increase could be used as a marker of a better nutritional state [3]. In fact, in a review from Akamatsu Y et al. [21], in a young and old population, the PhA was reported to be lower in women than in men, negatively correlated with age, and positively with BMI, MM, and HGS. In an older population with normal body weight, the PhA decreased in sarcopenic subjects and the prevalence of sarcopenia increased when the PhA was low [22]. On the other hand, it is well known that obesity is associated with lower PhA values, particularly in individuals with a BMI over 35 kg/m^2^. In our study population, the mean PhA value of 4.14° ± 0.83° was in accordance with previous findings [23].

A recent study in a population aged from 60 to 85 yrs with SO women registered lower PhA values [24] than both men (with and without sarcopenia) and No SO women. These differences were considered the expression of the sexual dimorphism of body composition, such as reduction in muscle mass (of about 25–45%) and lesser amounts of type I fiber in women than in men [25].

The phase angle has been studied as a prognostic marker in several clinical conditions and the possibility to monitor the progression of metabolic or cardiovascular risk factors with PhA changes in patients living with obesity is very promising. Few data are available on PhA changes during weight loss and contradictory results were observed with ketogenic enteral nutrition and VLKCD [5,26], independently of the range of weight loss. The preoperative PhA and HGS values can predict postoperative weight loss (%EWL) in patients following bariatric surgery procedures [27]. These measures reflect an increase in body cell mass (BCM) despite a loss of muscle mass, emphasizing the qualitative nature of HGS and the PhA as related measures. Furthermore, a recent meta-analysis by Campa et al. [7] demonstrated that resistance training promotes increases in the PhA, which result from an increase in extracellular resistance (Xc) concurrent with a reduction in intracellular resistance (Rz) in the older population.

Additionally, the group of patients with SO present a high frequency of a “Poor” score of QoL (about 50%) in both sexes. In a previous study, the QoL, evaluated with the SarQol questionnaire, is compromised in women with sarcopenia with a worsening score in those with sarcopenic obesity [28]. In these latter patients, no data are available regarding possible changes after weight loss. We observed that the whole PGWBI score had an improvement in the NoSO discharge patient group, but it was not linked to the probability of not being sarcopenic at discharge. This should be better investigated by also considering clinical complications and concomitant pharmacological therapies in future studies.

## 5. Conclusions

Following a 4-week multidisciplinary weight-loss program that combined a hypocaloric diet and physical activity, patients with sarcopenic obesity (SO) showed significant improvement. This improvement is primarily attributed to improvements in handgrip strength (HGS) values, with changes in the phase angle (PhA) further enhancing these effects. The PhA is a good indicator of better quality in skeletal muscle mass (SMM) and protein metabolism efficacy. Further research is needed to elucidate the predictive role of the PhA in SO under various nutritional interventions (e.g., supplementary foods, medical foods, very-low-calorie ketogenic diets, bariatric surgery, and new drug therapies) and physical interventions (e.g., aerobic exercise, anaerobic exercise, or their combinations). Additionally, these findings underscore the importance of tailored interventions in the effective management of SO, emphasizing the potential utility of the PhA as a valuable marker for treatment efficacy.

## Figures and Tables

**Table 1:** Clinical characteristics of SO patients at admission, by sex, and in whole cohort.

	Female (*N* = 129)	Male (*N* = 59)	All (*N* = 188)	*p*-Value
Age, years *mean* (*SD*)	64.8 (12.3)	63.5 (12.5)	64.4 (12.4)	0.432 ‡
Age class *N (%)*				
<65 yrs	55 (43%)	27 (32%)	82 (44%)	0.688 †
≥65 yrs	74 (57%)	32 (54%)	106 (56%)	
Weight, kg *mean* (*SD*)	96.1 (16.0)	113.2 (17.7)	101.5 (18.2)	**<0.001** ‡
BMI, kg/m^2^ *mean* (*SD*)	40.8 (6.0)	40.4 (5.4)	40.7 (5.8)	0.700 ‡
BMI class, *N (%)*				
BMI class I–II (30–40 kg/m^2^)	61 (47%)	32 (54%)	93 (49%)	0.376 †
BMI class III (>40 kg/m^2^)	68 (53%)	27 (46%)	95 (51%)	
PhA, ° *mean* (*SD*)	4.07 (0.82)	4.31 (0.83)	4.14 (0.83)	**0.048** ‡
FAT MASS, kg *mean* (*SD*)	49.5 (12.5)	47.5 (11.3)	48.8 (12.1)	0.318 ‡
FAT MASS, % *mean* (*SD*)	51.1 (4.8)	41.8 (4.9)	48.2 (6.5)	**<0.001** ¥
FAT-FREE MASS, kg *mean* (*SD*)	45.9 (5.3)	64.7 (8.7)	51.8 (10.9)	**<0.001** ‡
FAT-FREE MASS, % *mean* (*SD*)	48.4 (4.8)	57.7 (4.9)	51.3 (6.5)	**<0.001** ¥
MUSCLE MASS, kg *mean* (*SD*)	25.3 (4.1)	36.6 (6.1)	28.8 (7.1)	**<0.001** ‡
MUSCLE MASS, % *mean* (*SD*)	26.7 (4.1)	32.7 (4.2)	28.6 (5.0)	**<0.001** ¥
SMMI, % *mean* (*SD*)	19.1 (2.5)	26.0 (3.1)	21.3 (4.2)	**<0.001** ‡
HGS, kg *mean* (*SD*)	13.5 (1.7)	22.3 (3.8)	16.3 (4.8)	**<0.001** ‡
Waist, cm *mean* (*SD*)	129.05 (11.18)	117.21 (10.67)	120.94 (12.13)	**<0.001** ‡
PGWBI SCORE * *mean* (*SD*)	54.2 (20.4)	63.8 (21.5)		**0.009** ¥
PGWBI SCORE class * *N (%)*				
Good	21 (20%)	19 (40%)	40 (27%)	
Normal	25 (24%)	11 (23%)	36 (24%)	**0.026** †
Poor	57 (55%)	17 (36%)	74 (49%)	
Number of comorbidities	1.6 (1.1)	1.9 (1.1)	1.7 (1.1)	0.097 ‡

Data are expressed as mean and standard deviation (±SD) and number and/or percentage (%); BMI: body mass index; PhA, phase angle; SMMI, skeletal muscle mass index; HGS, handgrip strength; PGWBI, psychological general well-being index. ¥: *T*-test; ‡: Wilcoxon; †: Chi-square test; *: Data available for 150 patients out of the 188 with SO.

**Table 2:** Clinical characteristics of SO patients at admission in whole cohort and by SO status at discharge.

	No SO _discharge_ (*N* = 80)	SO _discharge_ (*N* = 108)	*p*-Value
Age, years, *mean* (*SD*)	63.11 (11.79)	65.36 (12.8)	0.125 ‡
Age class, *N (%)*			
<65 yrs	38 (48%)	44 (41%)	0.356 †
≥65 yrs	42 (52%)	64 (59%)	
Male, *N (%)*	22 (28%)	37 (34%)	0.323 †
Weight, kg *mean* (*SD*)	99.71 (17.69)	95.72 (15.85)	0.169 ‡
BMI, kg/m^2^ *mean* (*SD*)	39.46 (6.25)	38.82 (4.85)	0.724 ‡
BMI class, *N(%)*			
BMI class I–II (30–40 kg/m^2^)	39 (49%)	54 (50%)	0.865 †
BMI class III (>40 kg/m^2^)	42 (51%)	54 (50%)	
PhA, ° *mean* (*SD*)	4.18 (0.87)	4.06 (0.8)	0.564 ‡
FAT MASS, kg *mean* (*SD*)	49.45 (12.35)	45.55 (10.57)	**0.020** ‡
FAT MASS, % *mean* (*SD*)	48.91 (6.47)	47.12 (6.09)	0.054 ¥
FAT-FREE MASS, kg *mean* (*SD*)	50.77 (10.35)	50.39 (9.59)	0.940 ‡
FAT-FREE MASS, % *mean* (*SD*)	50.64 (6.46)	52.44 (6.06)	0.052 ¥
MUSCLE MASS, kg *mean* (*SD*)	28.39 (7.21)	27.75 (6.15)	0.733 ‡
MUSCLE MASS, % *mean* (*SD*)	28.34 (5.41)	28.94 (4.9)	0.425 ¥
SMMI, % *mean* (*SD*)	21.31 (4.12)	21.99 (4.21)	0.299 ‡
HGS, kg *mean* (*SD*)	21.97 (6.48)	16.13 (5.07)	**<0.0001** ‡
Waist, cm *mean* (*SD*)	122.27 (12.41)	119.93 (11.87)	**0.227** ‡
PGWBI SCORE * *mean* (*SD*)	74.17 (17.63)	68.06 (20.68)	0.060 ¥
PGWBI SCORE class * *N (%)*			
Good	17 (27%)	23 (26%)	
Normal	16 (25%)	20 (23%)	0.924 †
Poor	30 (48%)	44 (51%)	
Number of comorbidity	1.7 (1.2)	1.7 (1.0)	0.875 ‡

Data are expressed as mean and standard deviation (±SD) and number and/or percentage (%); BMI: body mass index; PhA, phase angle; SMMI, skeletal muscle mass index; HGS, handgrip strength; PGWBI, psychological general well-being index. ¥: *T*-test; ‡: Wilcoxon; †: Chi-square test; *: Data available for 150 patients out of the 188 with SO.

**Table 3:** Change in parameters (discharge–admission) in No SO discharge and SO discharge patients.

	No SO _discharge_ (*N* = 80)	SO _discharge_ (*N* = 108)	*p*-Value
Weight, kg *mean* (*SD*)	−4.49 (2.78)	−3.71 (2.32)	0.054 ‡
Weight, % *mean* (*SD*)	−4.15 (1.86)	−3.62 (1.91)	0.052 ‡
BMI, kg/m^2^ *mean* (*SD*)	−1.73 (0.97)	−1.48 (0.85)	0.088 ‡
PhA, ° *mean* (*SD*)	0.07 (0.9)	−0.11 (0.77)	0.094 ‡
FAT MASS, kg *mean* (*SD*)	−1.76 (3.14)	−1.53 (2.73)	0.515 ‡
FAT MASS, % *mean* (*SD*)	−0.36 (3)	−0.28 (2.28)	0.911 ‡
FAT-FREE MASS, kg *mean* (*SD*)	−1.31 (3.47)	−1.13 (2.46)	0.797 ‡
FAT-FREE MASS, % *mean* (*SD*)	0.36 (3)	0.33 (2.29)	0.990 ‡
MUSCLE MASS, kg *mean* (*SD*)	−0.65 (3.8)	−0.94 (3.15)	0.307 ‡
MUSCLE MASS, % *mean* (*SD*)	0.39 (3.86)	−0.18 (3.17)	0.265 ¥
SMMI, % *mean* (*SD*)	0.56 (2.03)	0.41 (1.52)	0.570 ¥
HGS, kg *mean* (*SD*)	5.46 (3.59)	0.05 (2.53)	**<0.0001** ‡
Waist, cm *mean* (*SD*)	−5.00 (4.52)	−5.61 (5.50)	0.375 ‡
PGWBI SCORE * *mean* (*SD*)	17.31 (16.95)	10.51 (13.11)	**0.005** ‡

Data are expressed as mean and standard deviation (±SD) and number and/or percentage (%); BMI: body mass index; PhA, phase angle; SMMI, skeletal muscle mass index; HGS, handgrip strength; PGWBI, psychological general well-being index. ¥: *T*-test; ‡: Wilcoxon; *: Data available for 150 patients out of the 188 with SO.

## Data Availability

Data described in the manuscript will be made available upon reasonable request.
